# The value of bronchodilator response in FEV1 and FeNO for differentiating between chronic respiratory diseases: an observational study

**DOI:** 10.1186/s40001-024-01679-w

**Published:** 2024-02-04

**Authors:** Zhaoqian Gong, Junwen Huang, Guiling Xu, Ying Chen, Maosheng Xu, Yanyan Ma, Wenqu Zhao, Yanhong Wang, Jianpeng Liang, Chunquan Ou, Laiyu Liu, Shaoxi Cai, Haijin Zhao

**Affiliations:** 1 Chronic Airways Diseases Laboratory, Department of Respiratory and Critical Care Medicine, Nanfang Hospital, Southern Medical University, Guangzhou, 510515 China; 2 Department of the Biostatistics, Guangdong Provincial Key Laboratory of Tropical Disease Research, School of Public Health, Southern Medical University, Guangzhou, China

**Keywords:** Chronic airway disease, ACO, Diagnosis, Bronchodilator response, Asthma–COPD overlap, Forced expiratory volume in 1 s, Fractional exhaled nitric oxide

## Abstract

**Background:**

There is no uniform standard for a strongly positive bronchodilation test (BDT) result. In addition, the role of bronchodilator response in differentiating between asthma, chronic obstructive pulmonary disease (COPD), and asthma–COPD overlap (ACO) in patients with a positive BDT result is unclear. We explored a simplified standard of a strongly positive BDT result and whether bronchodilator response combined with fractional exhaled nitric oxide (FeNO) can differentiate between asthma, COPD, and ACO in patients with a positive BDT result.

**Methods:**

Three standards of a strongly positive BDT result, which were, respectively, defined as post-bronchodilator forced expiratory volume in 1-s responses (ΔFEV_1_) increasing by at least 400 mL + 15% (standard I), 400 mL (standard II), or 15% (standard III), were analyzed in asthma, COPD, and ACO patients with a positive BDT result. Receiver operating characteristic curves were used to determine the optimal values of ΔFEV_1_ and FeNO. Finally, the accuracy of prediction was verified by a validation study.

**Results:**

The rates of a strongly positive BDT result and the characteristics between standards I and II were consistent; however, those for standard III was different. ΔFEV_1_ ≥ 345 mL could predict ACO diagnosis in COPD patients with a positive BDT result (area under the curve [AUC]: 0.881; 95% confidence interval [CI] 0.83–0.94), with a sensitivity and specificity of 90.0% and 91.2%, respectively, in the validation study. When ΔFEV_1_ was < 315 mL combined with FeNO < 28.5 parts per billion, patients with a positive BDT result were more likely to have pure COPD (AUC: 0.774; 95% CI 0.72–0.83).

**Conclusion:**

The simplified standard II can replace standard I. ΔFEV_1_ and FeNO are helpful in differentiating between asthma, COPD, and ACO in patients with a positive BDT result.

**Supplementary Information:**

The online version contains supplementary material available at 10.1186/s40001-024-01679-w.

## Background

Asthma and chronic obstructive pulmonary disease (COPD) are heterogeneous lung diseases [1], and they can coexist in some given patients, namely asthma–COPD overlap (ACO). Although some consensus documents raise different criteria for diagnosing ACO, there is still a lack of widely accepted and simplified criteria [2–4]. The European Consensus for ACO in 2016 is one of the most recognized criteria [4]. The prevalence of ACO in asthma and COPD is similar, ranging from 20 to 30% [5]. ACO has a higher symptom burden and more frequent and severe exacerbations than asthma or COPD [6, 7]. Additionally, the treatment of ACO is different from that of COPD; patients with ACO are recommended to use inhaled corticosteroids (ICS) combined with inhaled bronchodilators [8, 9] and might benefit from biologics used in patients with severe asthma [10, 11]. Therefore, it is of great importance to determine a simplified and accurate method for differentiating ACO from COPD.

The bronchodilation test (BDT), which evaluates airway reversibility, is not only an important diagnostic base of asthma, but it also plays a critical role in differentiating between ACO and COPD [12]. However, nearly one-third of patients with COPD have a positive BDT result [13–15]. The Global Initiative for Chronic Obstructive Lung Disease (GOLD) 2022 [16] also proposed that COPD alone often shows a positive BDT result when the baseline forced expiratory volume in 1 s (FEV_1_) is poor. As a major index for diagnosis of ACO according to the guidelines and consensus [4, 17–19], a strongly positive BDT result can be considered to differentiate ACO from COPD. Currently, there are three major criteria for a strongly positive BDT result: standard I [20–22], post-bronchodilator forced expiratory volume in 1 s response (ΔFEV_1_) > 400 mL + 15%; standard II [23], ΔFEV_1_ > 400 mL; and standard III [24], ΔFEV_1_ > 200 mL + 15%. However, it is unclear which one is more suitable in diagnosing ACO. Although the positive BDT result alone is limited in differentiating between asthma, COPD, and ACO, COPD has a lower ΔFEV_1_ (in mL) than asthma and ACO, and bronchodilator response (BDR) was helpful in the early screening of ACO [25]. Besides, BDR was vital in identifying different phenotypes of ACO [26]. Therefore, it is necessary to further explore the role of BDR in differentiating between asthma, COPD, and ACO in patients with a positive BDT result and determine whether there is a better predictive threshold.

The inflammatory biomarker fractional exhaled nitric oxide (FeNO) has been reported as an indicator for differentiating between ACO and COPD; one study reported its optimal cut-off value as 39.5 parts per billion (ppb) (sensitivity, 58.3%; specificity, 84.9%) [27], whereas another study reported its optimal cut-off value as 25.0 ppb (sensitivity, 60.6%; specificity, 87.7%) [28]. The two aforementioned studies showed a low sensitivity, indicating that FeNO alone is useful but difficult to use for differentiating between ACO and COPD. Recently, Wang reported that ΔFEV_1_ and FeNO were significantly different in ACO compared with COPD alone, which indicated that BDR combined with FeNO may be helpful in the early screening of ACO [25]. However, there is still a lack of an optimal value of ΔFEV_1_ for differentiating between asthma, COPD, and ACO in patients with a positive BDT result. It needs to be clarified whether ΔFEV_1_ combined with FeNO have advantages in differentiating between asthma, COPD, and ACO.

This study included patients with a positive BDT result to explore a simplified standard for a strongly positive BDT result and the value of ΔFEV_1_ in BDT and FeNO for differentiating between asthma, COPD, and ACO. We assumed that BDR in FEV_1_ and FeNO are helpful in differentiating between asthma, COPD, and ACO in patients with a positive BDT result, which contributes to the early screening of ACO.

## Methods

### Study design and patients

To explore a simplified standard for a strongly positive BDT result and the value of ΔFEV_1_, this cross-sectional study included patients admitted to our hospital’s outpatient respiratory clinic from January 2019 to January 2021. The participants were diagnosed with asthma, COPD, or ACO. The diagnosis of asthma was defined by the Global Initiative for Asthma (GINA) guidelines [29], requiring: (1) a history of asthmatic symptoms (wheezing, shortness of breath, with or without chest tightness or cough) relieved spontaneously or by medication; (2) variable expiratory airflow limitation (BDR of FEV_1_ > 200 mL and 12%). The diagnosis of COPD was based on the GOLD guidelines, indicating a post-bronchodilator FEV_1_/forced vital capacity (FVC) < 0.70 [30]. The diagnosis of ACO was based on the European Consensus 2016 criteria, major criteria [4]: (1) post-bronchodilator FEV_1_/FVC < 0.70 in individuals > 40 years old; (2) at least 10 pack-years of tobacco smoking or equivalent exposure history; (3) history of asthma before age of 40 years or BDR of FEV_1_ > 400 mL. Minor criteria included: (1) history of atopy or allergic rhinitis; (2) positive BDT result on two or more visits; (3) blood eosinophil count (BEC) ≥ 300 cells/μL. Patients who met all three major criteria and at least one minor criterion were diagnosed with ACO. Patients included were newly diagnosed and without inhalant treatment, or those who did not receive inhalant treatment for at least 4 weeks prior to enrollment, including long-acting muscarinic antagonist (LAMA), long-acting beta-2 agonist (LABA) orICS. All patients had a positive BDT result. The exclusion criteria were as follows: (1) acute attack of respiratory system; (2) active pulmonary tuberculosis, interstitial pneumonia, fungal infection and lung tumors; (3) refuse to sign informed consent. This study was approved by the ethics committee (Code No. NFEC-2021-142).

Clinical information from electronic medical records, including demographic data, spirometry data, BDT result, FeNO value, BEC, and percentage were collected. To verify the results, a validation study included patients who were admitted to the respiratory clinic from June 2021 to December 2022.

### Definition of the study groups

Three criteria were used to define a strongly positive BDT result in accordance with the GINA, National Institute for Health and Clinical Excellence, and American Thoracic Society (ATS) guidelines: standard I, ΔFEV_1_ > 400 mL + 15%; standard II, ΔFEV_1_ > 400 mL; and standard III, ΔFEV_1_ > 200 mL + 15%. Patients were grouped according to whether they had a strongly positive BDT result.

### Spirometry, BDT, FeNO, and BEC

Spirometry was strictly measured by spirometers (Jaeger MasterScreen, Germany) with reference to the ATS criterion. A positive BDT result was defined as follows: ΔFEV_1_ ≥ 200 mL + 12% after inhaling 400 μg of salbutamol. The FeNO detection was measured by a NIOX VERO analyzer (Aerocrine AB, Solna, Sweden) with reference to the ATS/European Respiratory Society criterion. The count and percentage of blood eosinophil was read by the automatic hematology analyzer.

### Statistical analysis

Statistical analysis was performed using SPSS statistics for Windows, version 24.0 (IBM Corp.). Data are presented as mean ± standard deviation for continuous variables and as median (first quartile, third quartile) for categorical variables. Comparisons between continuous variables were performed using the Student’s t-test or Mann–Whitney U test; the Chi-square test was used to analyze categorical variables. The factors of ΔFEV_1 _≥ 400 mL in ACO patients were analyzed using the COX regression model. All variables detected in the univariate analyses (with a *P*-value less than 0.1) were included in the multivariate analysis. Predictive values of single or combined measurements were calculated by constructing receiver operating characteristic (ROC) curves and measuring areas under the curve (AUCs). A two-sided *P*-value < 0.05 was considered significant.

## Results

### Study participants

**Fig. 1:**
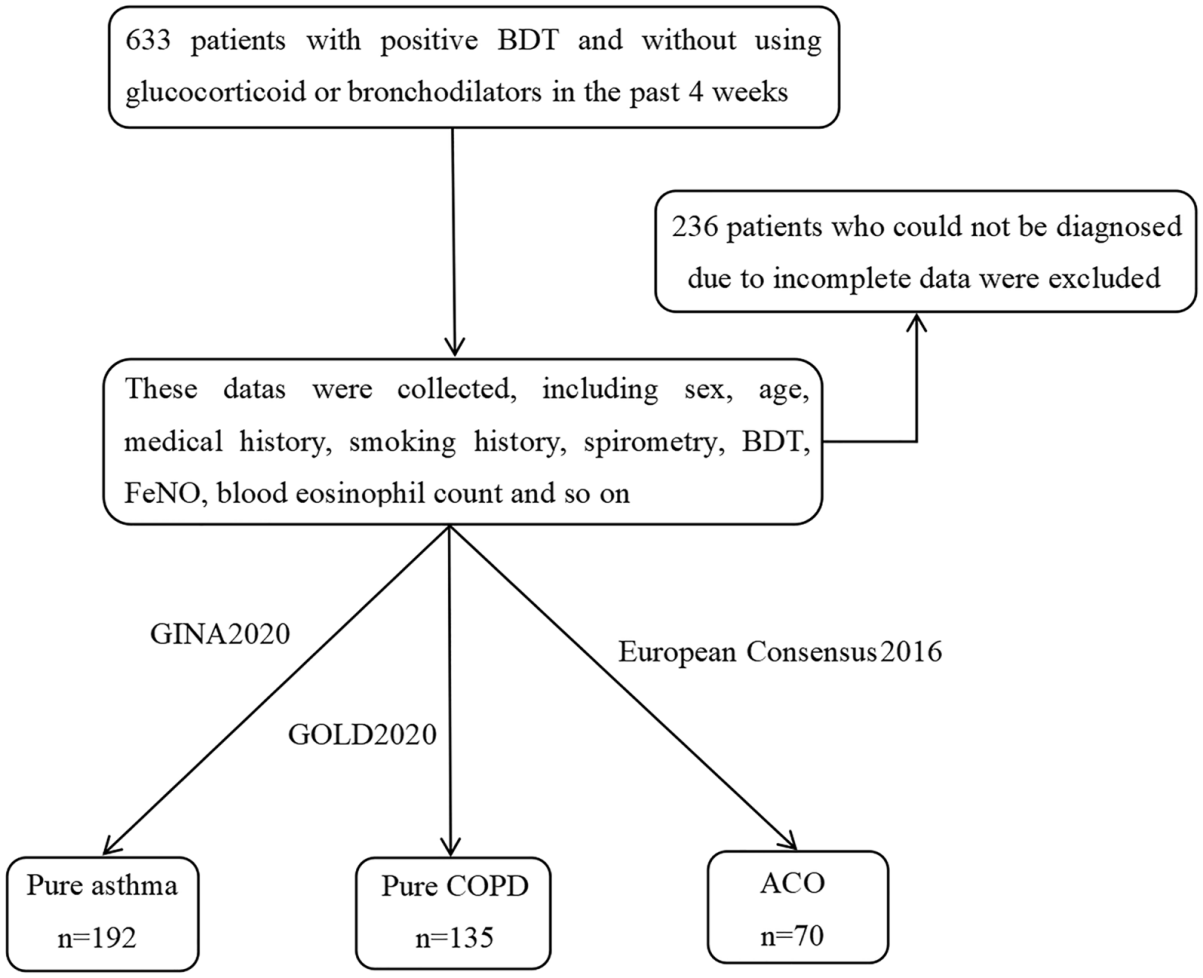
Study flowchart. BDT, bronchodilation test; FeNO, fractional exhaled nitric oxide; GINA, Global Initiative for Asthma; GOLD, Global Initiative for Chronic Obstructive Lung Disease; COPD, chronic obstructive pulmonary disease; ACO, asthma–chronic obstructive pulmonary disease overlap

A total of 633 patients were enrolled from the hospital’s outpatient respiratory clinic. Finally, only 397 patients were eligible, including 192 (48.4%) with asthma, 135 (34.0%) with COPD, and 70 (17.6%) with ACO. The study flow chart is shown in Fig. 1.

### Demographic and clinical characteristics

The clinical characteristics of the participants are shown in Table 1. Patients with asthma were younger, female-dominant, had lower smoking pack-years, and better spirometric indices (including FEV_1_, FVC, %FEV_1_, %FVC, and FEV_1_/FVC) than those with COPD or ACO. FEV_1_, FVC, %FEV_1_, and FEV_1_/FVC values of patients with COPD were lower than those of patients with ACO. Patients with ACO and asthma had a higher BDR in ΔFEV_1_ (mL) than those with COPD; the BDR in ΔFEV_1_ was highest in patients with ACO. Patients with asthma had a lower ΔFVC (mL) value than those with COPD and ACO; however, there was no difference in ΔFVC (mL) between patients with COPD and those with ACO. FeNO levels were lower in patients with COPD than in other patients, but there was no difference in FeNO levels between patients with asthma and those with ACO. The BEC was higher in patients with asthma than in those with COPD; however, there was no significant difference between patients with ACO and those with asthma or COPD. There was also no statistical difference in blood eosinophil percentage between patients with asthma, ACO, and COPD.

**Table 1:** Patient characteristics

	Asthma group (*N* = 192)	COPD group (*N* = 135)	ACO group (*N* = 70)	*P-*value
Age, year	46.0 (33.0, 55.5)	61.0 (56.0, 66.0)	56.0 (50.5, 62.3)	< 0.001
Sex (female/male), *N*	121/71	19/116	7/63	< 0.001
BMI, kg/m^2^	23.5 ± 3.5	22.9 ± 3.8	23.7 ± 2.9	0.178
*Smoking history*				
Current or ex-smoker/nonsmoker, *N*	38/154	111/24	53/17	< 0.001
Smoking pack-years	0.0 (0.0, 0.0)	30.0 (10.0, 40.0)	20.0 (0.0, 31.3)	< 0.001
Pulmonary function grading (normal/mild/moderate/moderate to severe/severe/extremely severe), *N*	32/70/37/26/21/7	0/19/30/22/41/23	0/23/17/9/12/9	< 0.001
*Post-bronchodilation spirometry*				
FEV_1_, L	1.91 (1.43, 2.44)	1.43 (1.02, 1.71)	1.75 (1.24, 2.08)	< 0.001
Predicted FEV_1_, %	69.7 ± 18.4	53.5 ± 18.1	61.7 ± 21.3	< 0.001
FVC, L	3.08 (2.53, 3.74)	2.90 (2.55, 3.34)	3.23 (2.55, 3.62)	0.004
Predicted FVC, %	96.5 ± 18.4	86.8 ± 17.9	90.4 ± 21.4	< 0.001
FEV_1_/FVC, %	60.7 ± 11.3	48.1 ± 11.6	53.9 ± 11.4	< 0.001
△FEV_1_, mL	340.0 (280.0, 480.0)	260.0 (230.0, 290.0)	425.0 (327.5, 530.0)	< 0.001
△FVC, mL	210.0 (92.5, 367.5)	300.0 (170.0, 470.0)	425.0 (117.5, 567.5)	< 0.001
Standard I, *N*(%)	66 (34.4)	4 (3.0)	40 (50.7)	< 0.001
Standard II, *N*(%)	74 (38.5)	4 (3.0)	42 (60)	< 0.001
Standard III, *N*(%)	135 (70.3)	103 (76.3)	61 (87.1)	0.019
FeNO, ppb	44.0 (17.0, 78.3)	21.0 (14.0, 51.0)	32.5 (20.8, 54.3)	0.003
*Blood parameters*				
Total eosinophils, /μL	340 (175, 493)	230 (130, 410)	255 (133, 465)	0.107
%Eosinophils	5.1 ± 3.5	3.9 ± 2.9	4.5 ± 3.4	0.251

Data are shown as frequency, mean ± SD, median (first quartile, third quartile), or frequency (percentage). COPD, chronic obstructive pulmonary disease; ACO, asthma–chronic obstructive pulmonary disease overlap; BMI, body mass index; FEV_1_, forced expiratory volume in 1 s; FVC, forced vital capacity; FeNO, fractional exhaled nitric oxide; SD, standard deviation

### Difference analysis of a strongly positive BDT rate under different standards

**Fig. 2:**
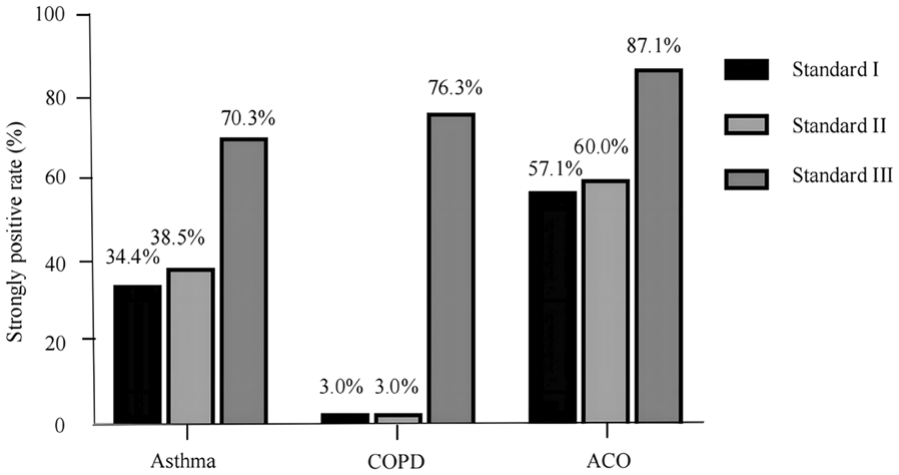
Strongly positive BDT rates in the asthma, COPD, and ACO groups under three different standards. COPD, chronic obstructive pulmonary disease; ACO, asthma–chronic obstructive pulmonary disease overlap; BDT, bronchodilation test

The strongly positive BDT rates in patients with asthma, COPD, and ACO under different standards are shown in Fig. 2 and Additional file 1. In the asthma group, 66 (34.4%), 74 (38.5%), and 135 (70.3%) patients had a strongly positive BDT rate under standards I, II, and III, respectively; those respective values were 4 (3.0%), 4 (3.0%), and 103 (76.3%) in the COPD group and 40 (57.1%), 42 (60.0%), and 61 (87.1%) in the ACO group. Under standards I and II, the ACO group had the highest strongly positive BDT rate, followed by the asthma group, and there were statistical differences between the three diseases (*P* < 0.05). Under standard III, the ACO group had a higher strongly positive BDT rate than the asthma group (*P* = 0.005), but there was no statistical difference between the COPD group and the other two groups. All three diseases had a higher strongly positive BDT rate in standard III than the other two standards (*P* < 0.001). However, there was no statistical difference in the strongly positive BDT rate between standards I and II among the three diseases (asthma, COPD, and ACO: *P* = 0.396, *P* = 1.000, and *P* = 0.731, respectively).

### Comparison of clinical characteristics under different standards

The comparison of clinical characteristics of asthma, COPD, and ACO under standards I, II, and III is shown in Additional files 2, 3 and 4, respectively. The clinical characteristics between the two groups with a strongly positive BDT result or not were consistent between standards I and II; however, clinical characteristics in standard III were different from those in standard I or II. Under standards I and II, asthma patients with a strongly positive BDT result were younger, more male-dominant, and had higher smoking pack-years and higher FEV_1_ and FVC values than those with COPD or ACO. Under any standard, there was no between-group difference in FeNO levels. However, under standard III, all patients with a strongly positive BDT result had poor pulmonary function, including FEV_1_, FVC, %FEV_1_, %FVC, and FEV_1_/FVC.

### Factors associated with ΔFEV_1_ ≥ 400 mL in ACO patients

The univariate and multivariate analysis with ΔFEV_1_ ≥ 400 mL in ACO patients is shown in Table 2. In the univariate Cox regression analysis, only FVC and ICS/LABA/LAMA (yes vs. no) were an independent predictor of ΔFEV_1_ ≥ 400 mL in ACO patients [FVC: HR = 1.97, 95% CI 1.04–3.71, *P* = 0.037; ICS/LABA/LAMA (yes vs. no): HR = 0.12, 95% CI 0.01–0.94, *P* = 0.044)]. The multivariate Cox regression analysis found that FVC was significantly correlated with ΔFEV_1_ ≥ 400 mL in ACO patients (HR = 2.71, 95% CI 1.31–5.63, *P* = 0.007). And the inhalation therapy of ICS/LABA/LAMA (yes vs. no) was also correlated with ΔFEV_1_ ≥ 400 mL in ACO patients (*P* = 0.013).

**Table 2:** Univariate and multivariate associations with ΔFEV_1_ ≥ 400 mL in ACO patients

Variable	Univariate analysis HR (95% Cl)	*P-*value	Multivariate analysis HR (95% Cl)	*P-*value
Age (year)	0.97 (0.92–1.02)	*P* = 0.187		
Sex (female vs. male)	1.40 (0.29–6.81)	*P* = 0.679		
BMI (kg/m^2^)	0.96 (0.81–1.13)	*P* = 0.598		
Smoking index (pack-year)	1.00 (0.97–1.02)	*P* = 0.765		
FEV_1_ (mL)	1.71 (0.77–3.78)	*P* = 0.188		
Predicted FEV_1_ (%)	1.01 (0.99–1.03)	*P* = 0.421		
FVC (mL)	1.97 (1.04–3.71)	*P* = 0.037	2.71 (1.31–5.63)	*P* = 0.007
Predicted FVC (%)	1.02 (0.99–1.04)	*P* = 0.132		
FEV_1_/FVC (%)	1.00 (0.96–1.05)	*P* = 0.982		
FeNO (ppb)	1.00 (0.99–1.02)	*P* = 0.519		
Total eosinophils (/μL)	1.00 (0.99–1.00)	*P* = 0.543		
Eosinophils (%)	0.99 (0.72–1.35)	*P* = 0.937	0.06 (0.01–0.55)	*P* = 0.013
ICS/LABA/LAMA (yes vs. no)	0.12 (0.01–0.94)	*P* = 0.044		

HR, relative risk; 95% CI, 95% confidence interval; BMI, body mass index; FEV_1_, forced expiratory volume in 1 s; FVC, forced vital capacity; FeNO, fractional exhaled nitric oxide; ICS, inhaled corticosteroids; LABA, long-acting beta-2 agonist; LAMA, long-acting muscarinic antagonist

### Predictive value of ΔFEV_1_ alone or combined with FeNO for the diagnosis of ACO or asthma in patients with a positive BDT result

**Fig. 3:**
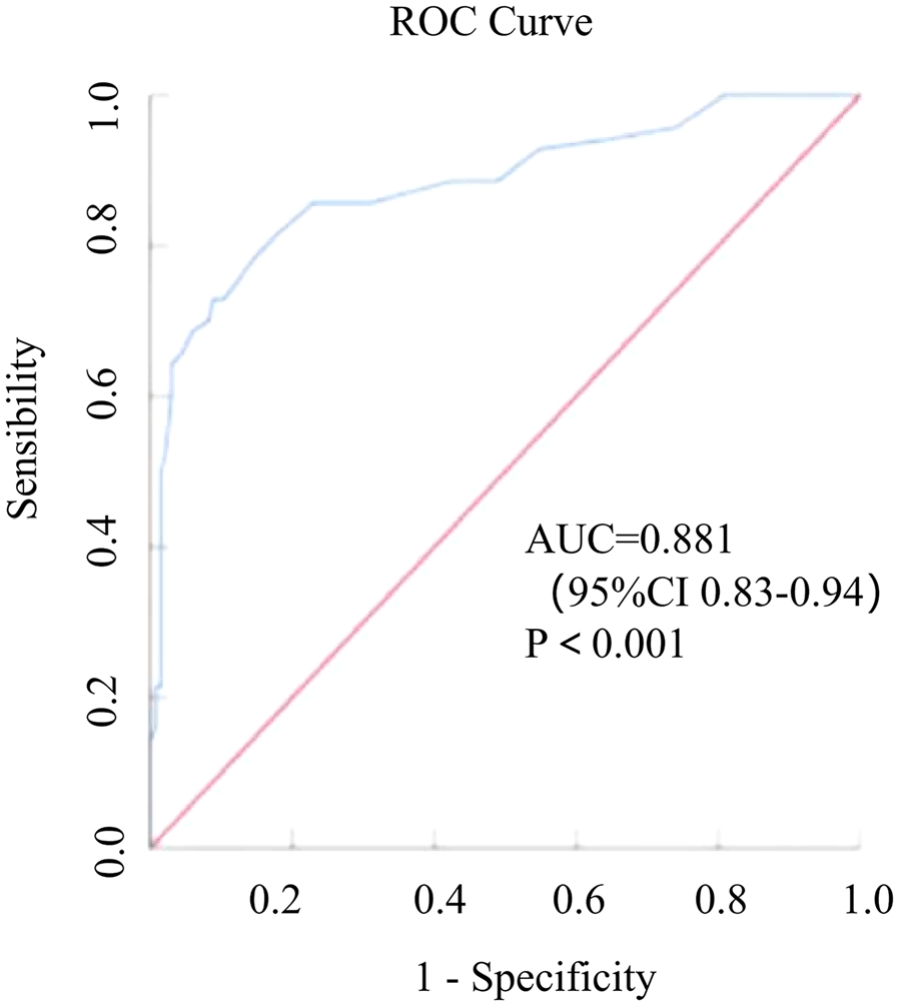
ROC curves for ΔFEV_1_ in predicting ACO diagnosis for COPD with a positive BDT result. ROC, receiver operating characteristic; ΔFEV_1_, post-bronchodilator forced expiratory volume in 1 s response; COPD, chronic obstructive pulmonary disease; ACO, asthma–chronic obstructive pulmonary disease overlap; BDT, bronchodilation test

The predictive value of ΔFEV_1_ alone or combined with FeNO was evaluated using ROC curves, adjusted by covariates. Only ΔFEV_1_ could predict the diagnosis of ACO in COPD patients with a positive BDT result, with a cut-off value of 345 mL (AUC: 0.881; 95% CI 0.83–0.94) (Fig. 3). Table 3 shows the sensitivity, specificity, positive predictive value, negative predictive value, and Youden index of each cut-off value for ΔFEV_1_.

**Table 3:** Predictive values for predicting ACO diagnosis from COPD in patients with a positive BDT result

ΔFEV_1_ (mL)	Sensibility (%)	Specificity (%)	PPV (%)	NPV (%)	Youden index
345	72.9	91.1	81.0	86.6	0.640
355	70.0	91.9	81.7	85.5	0.619
365	68.6	94.1	85.7	85.2	0.627
375	67.1	94.8	87.0	84.8	0.619
385	65.7	95.6	88.5	84.3	0.613

COPD, chronic obstructive pulmonary disease; ACO, asthma–chronic obstructive pulmonary disease overlap; BDT, bronchodilation test; FEV_1_, forced expiratory volume in 1 s; PPV, positive predictive values; NPV, negative predictive values

**Fig. 4:**
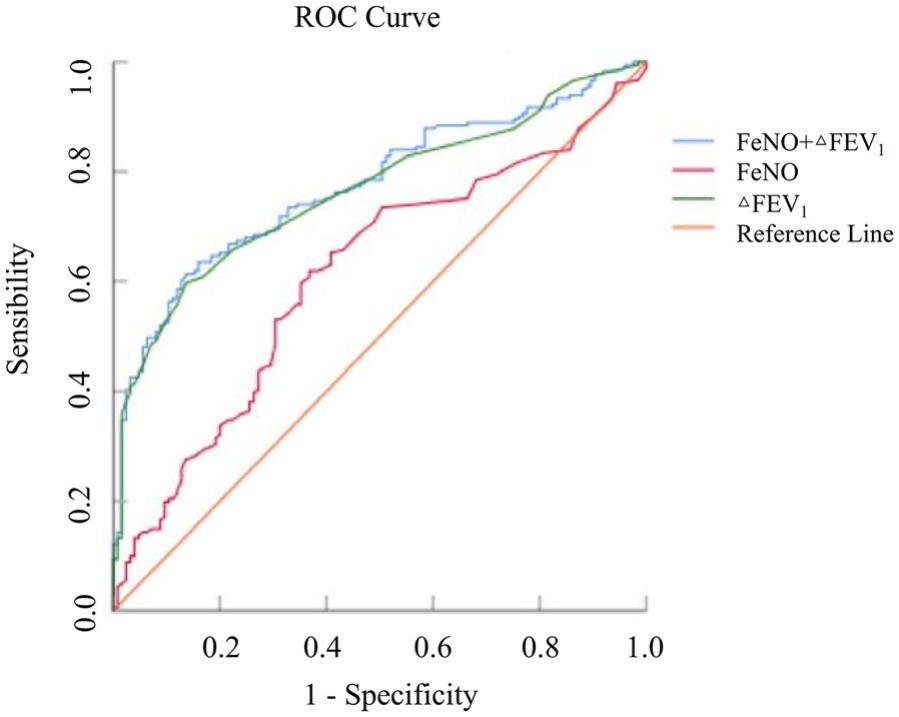
ROC curves for the model of ΔFEV_1_ combined with FeNO in predicting the diagnosis of asthma. ROC, receiver operating characteristic; ΔFEV_1_, post-bronchodilator forced expiratory volume in 1 s response; FeNO, fractional exhaled nitric oxide; BDT, bronchodilation test. ${\text{AUC}}_{{\text{FeNO}}+\Delta{\text{FEV}}_1} = 0.774$ (95% CI 0.72–0.83); AUC_FeNO_ = 0.613 (95% CI 0.55–0.68); ${\text{AUC}}_{\Delta{\text{FEV}}_1} = 0.765$ (95% CI 0.71–0.82)

After excluding patients with ACO, ΔFEV_1_ (AUC: 0.613; 95% CI 0.55–0.68) and FeNO (AUC: 0.765; 95% CI 0.71–0.82) could predict the diagnosis of asthma in patients with a positive BDT result (Table 4). The AUC for ΔFEV_1_ combined with FeNO was 0.774 (95% CI 0.72–0.83), which was significantly higher than that of ΔFEV_1_ or FeNO alone; the cut-off values for ΔFEV_1_ and FeNO were 315 mL and 28.5 ppb, respectively (Table 4 and Fig. 4).

**Table 4:** Predictive values for predicting asthma diagnosis in patients with a positive BDT result

	AUC	Cutoff value	Sensibility (%)	Specificity (%)	PPV (%)	NPV (%)	Youden index	*P*-value
FeNO (ppb)	0.613 (95% CI 0.55–0.68)	28.5	61.9	63.2	70.9	53.4	0.251	0.001
ΔFEV_1_ (mL)	0.765 (95% CI 0.71–0.82)	315	59.7	86.4	85.0	59.3	0.461	< 0.001
FeNO + ΔFEV_1_	0.774 (95% CI 0.72–0.83)	–	61.3	86.4	93.4	52.2	0.477	< 0.001

BDT, bronchodilation test; FeNO, fractional exhaled nitric oxide; FEV_1_, forced expiratory volume in 1 s; AUC, area under the curve; PPV, positive predictive values; NPV, negative predictive values

### Validation study

To verify the reliability of the prediction model, we continued to recruit 209 patients from June 2021 to December 2022, including 132 with asthma, 57 with COPD, and 20 with ACO. The clinical characteristics of the three diseases were broadly in line with our original study (Additional file 5). Surprisingly, ΔFEV_1_ ≥ 345 mL could predict the diagnosis of ACO in COPD patients with a positive BDT result, with a great sensitivity and specificity of 90.0% and 91.2%, respectively, in external validation (Additional file 6). Additionally, ΔFEV_1_ < 315 mL combined with FeNO < 28.5 ppb could eliminate asthma diagnosis in patients with a positive BDT result, with a high specificity of 87.0% but a low sensitivity of 54.2% (Additional file 7).

## Discussion

This is the first study to compare three criteria of a strongly positive BDT result in chronic airway disease patients with a positive BDT result. This study demonstrated that standard II (ΔFEV_1_ > 400 mL) can effectively replace standard I (ΔFEV_1_ > 400 mL + 15%).

In this study, patients with COPD were older, male-dominant, smoker-dominant, and had poorer baseline lung function than patients with asthma, which is consistent with previous studies’ findings [31, 32]. Compared with patients with COPD, those with ACO were younger and had better baseline lung function. A previous study reported that patients with ACO were younger than those with COPD, but they had a lower FEV_1_ [7]. Herein, patients with ACO and those with asthma had a higher BDR (mL in FEV_1_) than those with COPD, which is in line with previous studies’ findings [32, 33]. We found a significant difference in BECs between patients with asthma and those with COPD, but there was no difference between patients with ACO and those with COPD or asthma. We thought the reason for this phenomenon was that the positive BDT result and exposure to tobacco smoke reduced the difference in BECs between ACO and COPD or asthma [34–36]. Peng reported that patients with ACO had higher BECs than those with COPD [37]. However, there was no difference in BECs among the asthma, COPD, and ACO groups in the real-world study cohort, NOVELTY [6]. Additionally, patients with asthma and those with ACO had higher FeNO levels than those with COPD, which is similar to previous studies’ results [38, 39]. Therefore, BDR combined with the biomarkers of type 2 airway inflammation may be a useful tool in distinguishing between COPD and ACO or asthma.

A large multicenter study [40] reported that in 1106 participants with low FEV_1_ values (mL), the FEV_1_ increased by 12–44.7% relative to the baseline but < 200 mL, and ΔFEV_1_% increased with the level of airflow obstruction but decreased with severe obstruction, indicating that patients with severe obstruction rarely meet standard I. Thus, our result that standard II can replace standard I is clinically significant. However, there is still a lack of relevant research on the specific value of BDR in distinguishing between ACO and COPD with a positive BDT result.

Here, we firstly showed that ΔFEV_1_ ≥ 345 mL could help physicians to distinguish ACO from COPD in patients with a positive BDT result. To verify the accuracy of this conclusion, we strictly recruited 20 ACO, 57 COPD, and 132 asthma patients with a positive BDT result; we found that ΔFEV_1_ ≥ 345 mL was an excellent marker in distinguishing ACO from COPD. Similarly, a previous study showed that patients with ACO had a significantly higher ΔFEV_1_ value (mL) than those with COPD [25]. Moreover, some guidelines have suggested BDR ≥ 400 mL as the basis for distinguishing ACO from COPD, but in clinical practice, very few patients with COPD meet this criterion. It is very important to determine whether ΔFEV_1_ ≥ 345 mL could distinguish ACO from COPD in patients with a positive BDT result. Another study revealed that lung function parameters are potentially important tools in discriminating between asthma, ACO, and COPD [41]. COPD and asthma are characterized by incompletely reversible and reversible airflow obstruction, respectively [22, 42]. ACO shares the airflow obstruction characteristics of both asthma and COPD [22]. Thus, BDR is a key differential tool for distinguishing between COPD and ACO, especially in patients with a positive BDT result.

Alcázar-Navarrete [43] reported that an FeNO level of ≥ 19 ppb could distinguish ACO from COPD. Takayama [28] showed that COPD patients without treatment can be diagnosed as having ACO when the FeNO level is ≥ 25 ppb. In this study, patients with ACO had higher FeNO levels than those with COPD, but FeNO had no value in predicting ACO from COPD, which was inconsistent with previous studies’ findings [38, 44]. This result is likely due to the fact that patients had a positive BDT result, which can weaken the difference of the FeNO level between ACO and COPD. In the present study, most patients with a positive BDT result and airway limitation were diagnosed with COPD instead of asthma when ΔFEV_1_ < 315 mL was combined with an FeNO level < 28.5 ppb, which was verified by the validation study. This indicates that our prediction model is more meaningful in excluding a diagnosis.

This study has a few potential limitations, which should be considered. First, this is a single-center design, so multicenter studies are needed to confirm our findings. Secondly, all patients in this study are with a positive BDT result.

## Conclusions

Our study showed that the simplified standard II could replace the common standard I as the criterion of a strongly positive BDT result. Additionally, ΔFEV_1_ alone or combined with FeNO are helpful in differentiating between asthma, COPD, and ACO in patients with a positive BDT result.

### Supplementary Information

**Additional file 1**. Difference analysis of strongly positive asthma, COPD, and ACO rates under different standards.

**Additional file 2**. Clinical characteristics of different groups based on standard I.

**Additional file 3**. Clinical characteristics of different groups based on standard II.

**Additional file 4**. Clinical characteristics of different groups based on standard III.

**Additional file 5**. Patient characteristics in the validation study.

**Additional file 6**. The accuracy of ΔFEV_1_ ≥ 345 mL predicts the diagnosis of ACO from COPD with positive BDT.

**Additional file 7**. The accuracy of ΔFEV_1_ and FeNO in excluding asthma from patients with a positive BDT.

## Data Availability

The datasets used and/or analyzed during the current study are available from the corresponding author on reasonable request.

## Abbreviations

COPD: Chronic obstructive pulmonary disease
ACO: Asthma–chronic obstructive pulmonary disease overlap
BDT: Bronchodilation test
GOLD: Global Initiative for Chronic Obstructive Lung Disease
FEV_1_: Forced expiratory volume in 1 s
ΔFEV_1_: Postbronchodilator forced expiratory volume in 1-s response
FVC: Forced vital capacity
BDR: Bronchodilator response
FeNO: Fractional exhaled nitric oxide
GINA: Global initiative for asthma
BEC: Blood eosinophil count
ATS: American Thoracic Society
ROC: Receiver operating characteristic
AUC: Area under the curve
ICS: Inhaled corticosteroids
LABA: Long-acting beta-2 agonist
LAMA: Long-acting muscarinic antagonist
HR: Relative risk
CI: Confidence interval
