# Supplementary material for: The value of bronchodilator response in FEV1 and FeNO for differentiating between chronic respiratory diseases: an observational study
Source: Eur J Med Res. 2024 Feb 4;29:97. doi: 10.1186/s40001-024-01679-w (PMC10840153; doi:10.1186/s40001-024-01679-w)
Supplement: Supplementary file 1 — Additional file 1. Difference analysis of strongly positive asthma, COPD, and ACO rates under different standards. [file 40001_2024_1679_MOESM1_ESM.pdf]

Additional File 1. Difference analysis of strongly positive asthma, COPD, and ACO rates under different standards.

	Standard I	Standard II	Standard III	P value
	Strongly positive+	Strongly positive+	Strongly positive+	
Asthma n (%)	66 (34.4)	74 (38.5)	135 (70.3)	**< 0.001**
COPD n (%)	4 (3.0)	4 (3.0)	103 (76.3)	**< 0.001**
ACO n (%)	40 (57.1)	42 (60.0)	61 (87.1)	**< 0.001**

Data are shown as frequency (percentage). COPD, chronic obstructive pulmonary disease; ACO, asthma-chronic obstructive pulmonary disease overlap; Strongly positive+, strongly positive bronchodilation test
