# Supplementary material for: The value of bronchodilator response in FEV1 and FeNO for differentiating between chronic respiratory diseases: an observational study
Source: Eur J Med Res. 2024 Feb 4;29:97. doi: 10.1186/s40001-024-01679-w (PMC10840153; doi:10.1186/s40001-024-01679-w)
Supplement: Supplementary file 2 — Additional file 2. Clinical characteristics of different groups based on standard I. [file 40001_2024_1679_MOESM2_ESM.pdf]

Additional File 2. Clinical characteristics of different groups based on standard I.

	Asthma group (N=192)		P value	COPD group (N=135)		P value	ACO group (N=70)		P value
	Strongly positive+ (N=66)	Strongly positive- (N=126)		Strongly positive+ (N=4)	Strongly positive- (N=131)		Strongly positive+ (N=40)	Strongly positive- (N=30)	
Age , year	39 ± 13	47 ± 13	**<0.001**	61 ± 8	62 ± 9	0.87	56 ± 11	58 ± 11	0.52
Sex(female/male), N	27/39	94/32	**<0.001**	1/3	18/113	0.52	4/36	3/27	1.00
BMI, kg/m^2	23.3 ± 4.2	26.9 ± 36.2	0.42	25.6 ± 4.2	22.8 ± 3.7	0.15	23.6 ± 3.0	24.0 ± 2.8	0.58
Smoking History									
Current or ex-smoker /nonsmoker, N	23/43	11/115	**<0.001**	3/1	108/23	0.70	28/12	25/5	0.20
Smoking pack-years	0 (0, 5)	0 (0, 0)	**<0.001**	16 ± 19	29 ± 25	0.28	22 ± 22	22 ± 18	0.95
Pulmonary function grading									
(normal/mild/moderate/moderate to severe /severe/extremely severe), N	6/21/20/9/7/3	25/49/17/17/14 /4	0.07	0/0/1/1/2/0	0/19/29/21/39/ 23	0.73	0/14/11/5/5/5	0/9/6/4/7/4	0.79
Post-bronchodilation spirometry									
FEV_1, L	2.15 ± 0.69	1.83 ± 0.64	**0.001**	1.67 ± 0.83	1.40 ± 0.48	0.29	1.81 ± 0.58	1.69 ± 0.72	0.45
Predicted FEV_1, %	67.0 ± 16.5	71.1 ± 19.2	0.15	59.9 ± 16.2	53.3 ± 18.1	0.48	63.0 ± 19.3	60.0 ± 24.0	0.56
FVC, L	3.62 ± 0.89	2.94 ± 0.80	**<0.001**	2.56 ± 0.65	2.91 ± 0.68	0.32	3.34 ± 0.84	3.04 ± 0.90	0.17
Predicted FVC, %	95.9 (85.6, 101.5)	94.8 (85.0, 111.3)	0.37	79.3 ± 6.1	87.0 ± 18.1	0.40	93.1 ± 19.9	86.8 ± 23.0	0.22
FEV_1/FVC, %	58.8 ± 10.8	61.7 ± 11.5	0.09	51.7 ± 13.4	48.0 ± 11.6	0.53	53.9 ± 10.6	54.0 ± 12.6	0.97
FeNO, ppb	56.6 ± 41.8	45.5 ± 42.9	0.09	18.3 ± 8.5	35.3 ± 31.6	0.29	36.0 (21.5, 63.0)	24.0 (15.0, 92.0)	0.67
Blood parameters									
Total eosinophil, /µl	450 (308, 638)	315 (118, 485)	0.33	*	*	*	210(120, 400)	310(130, 590)	0.57
%Eosinophils	6.1 ± 2.6	4.7 ± 3.8	0.19	*	*	*	4.5 ± 3.6	4.6 ± 3.3	0.94

Data are shown as frequency, mean ± SD, median (first quartile, third quartile), or frequency (percentage). "*", the data were insufficient for analysis.

COPD, chronic obstructive pulmonary disease; ACO, asthma-chronic obstructive pulmonary disease overlap; Strongly positive+, strongly positive bronchodilation test; Strongly positive-, non-strongly positive bronchodilation test; BMI, body mass index; FEV1, forced expiratory volume in 1 second; FVC, forced vital capacity; FeNO, fractional exhaled nitric oxide. SD, standard deviation
