# Supplementary material for: The value of bronchodilator response in FEV1 and FeNO for differentiating between chronic respiratory diseases: an observational study
Source: Eur J Med Res. 2024 Feb 4;29:97. doi: 10.1186/s40001-024-01679-w (PMC10840153; doi:10.1186/s40001-024-01679-w)
Supplement: Supplementary file 3 — Additional file 3. Clinical characteristics of different groups based on standard II. [file 40001_2024_1679_MOESM3_ESM.pdf]

Additional File 3. Clinical characteristics of different groups based on standard II.

	Asthma group (N=192)		P value	COPD group (N=135)		P value	ACO group (N=70)		P value
	Strongly positive+ (N=74)	Strongly positive- (N=118)		Strongly positive+ (N=4)	Strongly positive- (N=131)		Strongly positive+ (N=42)	Strongly positive- (N=28)	
Age, year	38 ± 13	48 ± 13	**<0.001**	61 ± 9	62 ± 9	0.87	55 ± 11	59 ± 10	0.20
Sex (female/male), N	28/46	93/25	**<0.001**	1/3	18/113	0.52	4/38	3/25	0.87
BMI, kg/m^2	23.2 ± 4.0	27.2 ± 37.4	0.36	25.6 ± 4.2	22.8 ± 3.7	0.15	23.6 ± 2.9	23.9 ± 2.9	0.64
Smoking History									
Current or ex-smoker/ nonsmoker, N	28/46	10/108	**<0.001**	3/1	108/23	0.70	30/12	23/5	0.31
Smoking pack-years	0 (0, 5)	0 (0, 0)	**<0.001**	16 ± 19	29 ± 25	0.28	22 ± 22	22 ± 18	0.99
Pulmonary function grading (normal/mild/moderate/moderate to severe/ severe/extremely severe), N	9/25/21/9/7/3	22/45/16/17/ 14/4	0.22	0/0/1/1/2/0	0/19/29/21/39/ 23	0.73	0/16/11/5/5/5	0/7/6/4/7/4	0.58
Post-bronchodilation spirometry									
FEV_1, L	2.23(1.69, 2.80)	1.77(1.39, 2.07)	**<0.001**	1.67 ± 0.83	1.40 ± 0.48	0.29	1.87 ± 0.64	1.58 ± 0.62	0.06
Predicted FEV_1, %	69.0 ± 17.1	70.1 ± 19.1	0.71	59.9 ± 16.2	53.3 ± 18.1	0.48	64.6 ± 20.1	57.4 ± 22.7	0.17
FVC, L	3.70(3.03, 4.44)	2.81(2.40, 3.30)	**<0.001**	2.56 ± 0.65	2.91 ± 0.68	0.32	3.42 ± 0.90	2.90 ± 0.75	**0.02**
Predicted FVC, %	95.0(86.1, 102.6)	94.9(84.5, 111.5)	0.62	79.3 ± 6.1	87.0 ± 18.1	0.40	94.4 ± 20.3	84.4 ± 21.8	0.06
FEV_1/FVC, %	60.1 ± 11.1	61.1 ± 11.5	0.56	51.7 ± 13.4	48.0 ± 11.6	0.53	54.5 ± 10.8	53.1 ± 12.4	0.61
FeNO, ppb	65.0(36.5, 112.0)	36.0(14.0, 73.0)	**0.02**	18.3 ± 8.5	35.3 ± 31.6	0.29	36.0(21.5, 63.0)	24.0(15.0, 92.0)	0.40

Blood parameters									
Total eosinophils, /μl	430(275, 530)	330(125, 495)	0.19	*	*	*	210(120, 400)	310(130, 590)	0.57
%Eosinophils	5.6 ± 2.7	4.8 ± 3.9	0.43	*	*	*	4.5 ± 3.6	4.6 ± 3.3	0.94

Data are shown as frequency, mean ± SD, median (first quartile, third quartile), or frequency (percentage). "*", the data were insufficient for analysis. COPD, chronic obstructive pulmonary disease; ACO, asthma-chronic obstructive pulmonary disease overlap; Strongly positive+, strongly positive bronchodilation test; Strongly positive-, non-strongly positive bronchodilation test; BMI, body mass index; FEV1, forced expiratory volume in 1 second; FVC, forced vital capacity; FeNO, fractional exhaled nitric oxide. SD, standard deviation
