# Supplementary material for: The value of bronchodilator response in FEV1 and FeNO for differentiating between chronic respiratory diseases: an observational study
Source: Eur J Med Res. 2024 Feb 4;29:97. doi: 10.1186/s40001-024-01679-w (PMC10840153; doi:10.1186/s40001-024-01679-w)
Supplement: Supplementary file 4 — Additional file 4. Clinical characteristics of different groups based on standard III. [file 40001_2024_1679_MOESM4_ESM.pdf]

Additional File 4. Clinical characteristics of different groups based on standard III.

	Asthma group (N=192)		P value	COPD group (N=135)		P value	ACO group (N=70)		P value
	Strongly positive+ (N=135)	Strongly positive- (N=57)		Strongly positive+ (N=103)	Strongly positive- (N=32)		Strongly positive+ (N=61)	Strongly positive- (N=9)	
Age, year	47 (32, 56)	45 (34, 52)	0.59	63 ± 8	60 ± 10	0.08	57 ± 11	56 ± 10	0.96
Sex (female/male), N	82/53	39/18	0.31	17/86	2/30	0.15	7/54	0/9	0.28
BMI, kg/m^2	23.6 ± 3.6	30.5 ± 53.7	0.14	22.5 ± 3.7	24.2 ± 3.6	**0.03**	23.7 ± 3.0	23.6 ± 2.6	0.92
Smoking History									
Current or ex-smoker/ nonsmoker, N	26/109	12/45	0.78	84/19	27/5	0.72	44/17	9/0	0.07
Smoking pack-years	2 ± 6	1 ± 3	0.30	30 ± 25	25 ± 22	0.30	21 ± 21	30 ± 19	0.23
Pulmonary function grading (normal/mild/moderate/moderate to severe/ severe/extremely severe), N	14/40/30/24/20/7	17/30/7/2/1/0	**<0.001**	0/9/19/13/39/23	0/10/11/9/2/0	**<0.001**	0/16/15/9/12/9	0/7/2/0/0/0	**0.03**
Post-bronchodilation spirometry									
FEV_1, L	1.79 ± 0.66	2.30 ± 0.57	**<0.001**	1.26 ± 0.41	1.91 ± 0.38	**<0.001**	1.66 ± 0.60	2.41 ± 0.53	**0.001**
Predicted FEV_1, %	63.9(54.3, 75.8)	82.7(74.0, 88.6)	**<0.001**	49.2 ± 17.1	67.4 ± 13.5	**<0.001**	58.4 ± 20.3	84.2 ± 14.1	**<0.001**
FVC, L	3.09 ± 0.92	3.38 ± 0.77	**0.04**	2.76 ± 0.65	3.34 ± 0.56	**<0.001**	3.13 ± 0.86	3.75 ± 0.82	**0.05**
Predicted FVC, %	93.8 ± 18.6	102.9 ± 16.2	**0.001**	84.7 ± 17.9	93.7 ± 16.2	**0.012**	88.4±21.1	104.0 ± 18.5	**0.04**
FEV_1/FVC, %	58.8(50.3, 65.9)	69.1(63.2, 74.0)	**<0.001**	43.6(37.8, 52.8)	58.9(50.3, 63.9)	**<0.001**	52.2 ± 11.0	65.3 ± 6.4	**0.001**
FeNO, ppb	37.0(17.0, 73.8)	64.5(18.8, 104.5)	0.67	21.0(13.8, 41.3)	34.0(11.5, 87.0)	0.88	48.1 ± 42.2	45.6 ± 47.7	0.88
Blood parameters									

Total eosinophils, /µl	365 (175, 513)	330 (115, 448)	0.51	250(145, 343)	220 (65, 645)	0.38	306 ± 200	315±233	0.95
%Eosinophils	5.1 ± 3.2	5.2 ± 4.4	0.93	3.9 ± 2.9	4.0 ± 3.4	0.93	4.6 ± 3.6	4.0±2.2	0.82

Data are shown as frequency, mean ± SD, median (first quartile, third quartile), or frequency (percentage). COPD, chronic obstructive pulmonary disease; ACO, asthma-chronic obstructive pulmonary disease overlap; Strongly positive+, strongly positive bronchodilation test; Strongly positive-, non-strongly positive bronchodilation test; BMI, body mass index; FEV1, forced expiratory volume in 1 second; FVC, forced vital capacity; FeNO, fractional exhaled nitric oxide. SD, standard deviation
