# Supplementary material for: The value of bronchodilator response in FEV1 and FeNO for differentiating between chronic respiratory diseases: an observational study
Source: Eur J Med Res. 2024 Feb 4;29:97. doi: 10.1186/s40001-024-01679-w (PMC10840153; doi:10.1186/s40001-024-01679-w)
Supplement: Supplementary file 5 — Additional file 5. Patient characteristics in the validation study. [file 40001_2024_1679_MOESM5_ESM.pdf]

Additional File 5. Patient characteristics in the validation study.

	Asthma group (N=132)	COPD group (N=57)	ACO group (N=20)	P value
Age, year	45.2 ± 15.0	61.1 ± 6.3	56.7 ± 8.8	**<0.001**
Sex (famale/male), N	59/73	5/52	0/20	**<0.001**
BMI, kg/m^2	23.6 ± 3.2	22.4 ± 3.4	23.7 ± 3.8	0.062
Smoking History				
Current or ex-smoker/nonsmoker, N	46/86	49/8	19/1	**<0.001**
Smoking pack-years	0 (0, 5)	40 (26, 50)	30 (20, 40)	**<0.001**
Pulmonary function grading (normal/mild/moderate/moderate to severe/severe/extremely severe), N	10/64/20/21/10/7	0/7/9/14/14/13	0/8/1/2/5/4	**<0.001**
Past-bronchodilation spirometry				
FEV1, L	2.02 ± 0.74	1.34 ± 0.45	1.74 ± 0.67	**<0.001**
Predicted FEV1, %	69.3 ± 18.2	50.3 ± 15.1	56.5 ± 20.2	**<0.001**
FVC, L	3.20 ± 0.10	2.8 ± 0.7	3.5 ± 0.9	**0.013**
Predicted FVC, %	90.9 ± 15.9	83.3 ± 15.5	90.1 ± 19.0	**0.020**
FEV1/FVC, %	62.6 ± 11.8	46.7 ± 9.4	49.0 ± 10.2	**<0.001**
△FEV1, mL	345.0 (282.5, 457.5)	260.0 (230.0, 300.0)	415.0 (362.5, 475.0)	**<0.001**
△FVC, mL	210.0 (102.5, 350.0)	290.0 (200.0, 465.0)	355.0 (242.5, 677.5)	**<0.001**
Standard I, N (%)	36 (27.3)	2 (3.5)	12 (60)	**<0.001**
Standard II, N (%)	48 (36.4)	2 (3.5)	12 (60)	**<0.001**

Standard III, N (%)	85 (64.4)	47 (82.5)	19 (95.0)	**0.002**
FeNO, ppb	65.6 ± 63.7	32.6 ± 26.7	71.2 ± 55.0	**0.001**
**Blood parameters**				
Total eosinophils, /µl	329 ± 289	355 ± 330	290 ± 180	0.955
%Eosinophils	4.1 ± 3.3	4.3 ± 3.8	4.9 ± 3.3	0.781

Data are shown as frequency, mean ± SD, median (first quartile, third quartile), or frequency (percentage). COPD, chronic obstructive pulmonary disease; ACO, asthma-chronic obstructive pulmonary disease overlap; BMI, body mass index; FEV1, forced expiratory volume in 1 second; ΔFEV1, postbronchodilator forced expiratory volume in 1 second response; FVC, forced vital capacity; ΔFVC, postbronchodilator forced vital capacity response; FeNO, fractional exhaled nitric oxide; SD, standard deviation
