# Supplementary material for: The value of bronchodilator response in FEV1 and FeNO for differentiating between chronic respiratory diseases: an observational study
Source: Eur J Med Res. 2024 Feb 4;29:97. doi: 10.1186/s40001-024-01679-w (PMC10840153; doi:10.1186/s40001-024-01679-w)
Supplement: Supplementary file 6 — Additional file 6. The accuracy of ΔFEV1 ≥ 345 mL predicts the diagnosis of ACO from COPD with positive BDT. [file 40001_2024_1679_MOESM6_ESM.pdf]

Additional File 6. The accuracy of ΔFEV1≥345 mL predicts the diagnosis of ACO from COPD with positive BDT.

$\Delta FEV_1 \geq$ 345mL	ACO	COPD	Total
Yes	18	5	23
No	2	52	54
Total	20	57	77

ΔFEV1, postbronchodilator forced expiratory volume in 1-second response; COPD, chronic obstructive pulmonary disease; ACO, asthma-chronic obstructive pulmonary disease overlap; BDT, bronchodilation test
