# Supplementary material for: The value of bronchodilator response in FEV1 and FeNO for differentiating between chronic respiratory diseases: an observational study
Source: Eur J Med Res. 2024 Feb 4;29:97. doi: 10.1186/s40001-024-01679-w (PMC10840153; doi:10.1186/s40001-024-01679-w)
Supplement: Supplementary file 7 — Additional file 7. The accuracy of ΔFEV1 and FeNO in excluding asthma from patients with a positive BDT. [file 40001_2024_1679_MOESM7_ESM.pdf]

Additional File 7. The accuracy of ΔFEV1 and FeNO in excluding asthma from patients with a positive BDT.

ΔFEV_1 < 315 mL + FeNO < 28.5 ppb	COPD	Asthma	Total
Yes	26	17	43
No	22	114	136
Total	48	131	179

ΔFEV1, postbronchodilator forced expiratory volume in 1-second response; FeNO, fractional exhaled nitric oxide; COPD, chronic obstructive pulmonary disease; BDT, bronchodilation test
